# Diagnosis of tuberculosis infection in pediatric patients treated with inhibitors of the tumour necrosis factor alpha. A multicenter national study comparing tuberculin skin test and igra tests

**DOI:** 10.1186/1546-0096-12-S1-P282

**Published:** 2014-09-17

**Authors:** Antoni Noguera Julian, Jordi Antón López, Joan Calzada-Hernández, Esmeralda Núñez Cuadros, Maria José Mellado Peña, Francisco Javier Martín Carpi

**Affiliations:** 1 Pediatric Infectious Diseases Unit, Pediatrics Department, Hospital Sant Joan de Déu, Esplugues de Llobregat (Barcelona), Spain; 2 Pediatric Rheumatology Unit, Pediatrics Department, Hospital Sant Joan de Déu, Esplugues de Llobregat (Barcelona), Spain; 3 Hospital Regional Universitario de Málaga (Hospital Carlos Haya), Málaga, Spain; 4 Hospital Universitario La Paz, Madrid, Pediatric Gastroenterology Department, Hospital Sant Joan de Déu, Esplugues de Llobregat (Barcelona), Spain; 5 TBC

## Introduction

In the last years, inhibitors of tumor necrosis factor alpha (antiTNFα) have been a major advance in the treatment of many rheumatic diseases and inflammatory bowel disease, also in the pediatric patient. However, antiTNFα use is associated with an increased risk of serious infections, including tuberculosis (TB). In adults, the new interferon γ release assays (IGRA) tests for the diagnosis of TB infection appear to show better sensitivity and specificity than the tuberculin skin test (TST) in these patients. Data in children are still very scarce. We have previously reported a latent tuberculosis infection (LTI) prevalence rate of 1.4% (95%CI: 0-2.9) in children on antiTNFα treatment, which is similar to that reported in healthy pediatric population studies in Spain. LTI was diagnosed in 3 adolescent girls (out of 221 patients) in whom QTF tested positive, while TST was positive in only one of them.

## Objectives

To establish the sensitivity and specificity of IGRA tests in the diagnosis of LTI compared to the TST in pediatric patients by the implementation of antiTNFα treatment.

## Methods

We present an ongoing national multicenter retrospective/prospective cross-sectional study (hosted by the Spanish National Societies of Pediatric Rheumatology, Infectious Diseases and Gastroenterology) including pediatric patients in whom LTI is to be ruled out simultaneously by TST and IGRA test, prior to initiating treatment with antiTNFα drugs. Medical history related to the underlying disease, the risk of LTI and the results of the TST and IGRA tests will be collected.

## Conclusion

This study will observe the prevalence of LTI in this cohort, identify risk factors associated with LTI and analyze the sensitivity, specificity and negative predictive value of IGRA tests as compared to TST in children before antiTNFα initiation.

## Disclosure of interest

None declared.

